# Legal and institutional foundations for universal health coverage, Kenya

**DOI:** 10.2471/BLT.19.237297

**Published:** 2020-09-03

**Authors:** Regina Mbindyo, Jackson Kioko, Fred Siyoi, Stephen Cheruiyot, Mary Wangai, Joyce Onsongo, Annette Omwoyo, Christine Kisia, Koome Miriti

**Affiliations:** a World Health Organization Country Office, UN Complex Gigiri, Block U3, UN Gigiri Avenue, Nairobi, Kenya.; b Kenya Health Professions Oversight Authority, Nairobi, Kenya.; c Pharmacy and Poisons Board, Nairobi, Kenya.; d Ministry of Health, Nairobi, Kenya.; e Kenya Law Reform Commission, Nairobi, Kenya.; f Kenya National Commission on Human Rights, Nairobi, Kenya.

## Abstract

Kenya’s Constitution of 2010 triggered a cascade of reforms across all sectors to align with new constitutional standards, including devolution and a comprehensive bill of rights. The constitution acts as a platform to advance health rights and to restructure policy, legal, institutional and regulatory frameworks towards reversing chronic gaps and improving health outcomes. These constitutionally mandated health reforms are complex. All parts of the health system are transforming concurrently, with several new laws enacted and public health bodies established. Implementing such complex change was hampered by inadequate tools and approaches. To gain a picture of the extent of the health reforms over the first 10 years of the constitution, we developed an adapted health-system framework, guided by World Health Organization concepts and definitions. We applied the framework to document the health laws and public bodies already enacted and currently in progress, and compared the extent of transformation before and after the 2010 Constitution. Our analysis revealed multiple structures (laws and implementing public bodies) formed across the health system, with many new stewardship structures aligned to devolution, but with fragmentation within the regulation sub-function. By deconstructing normative health-system functions, the framework enabled an all-inclusive mapping of various health-system attributes (functions, laws and implementing bodies). We believe our framework is a useful tool for countries who wish to develop and implement a conducive legal foundation for universal health coverage. Constitutional reform is a mobilizing force for large leaps in health institutional change, boosting two aspects of feasibility for change: stakeholder acceptance and authority to proceed.

## Résumé

Adoptée en 2010, la Constitution du Kenya a entraîné une série de réformes dans tous les secteurs afin de les adapter aux nouvelles normes constitutionnelles, notamment à la décentralisation et à une charte détaillée des droits. La Constitution sert de tremplin pour faire progresser les droits en matière de santé et restructurer les cadres politiques, juridiques, institutionnels et réglementaires en vue de réduire les disparités chroniques et d'améliorer les résultats cliniques. Toutefois, ces réformes de santé prévues dans la Constitution sont complexes. Toutes les composantes du système de santé évoluent en même temps, de nombreuses lois inédites sont promulguées et des organismes de santé publique sont créés. L'emploi d'approches et d'outils inadaptés a entravé la mise en œuvre de ces changements si complexes. Pour mieux appréhender l'étendue des réformes de santé entreprises au cours des 10 premières années de la Constitution, nous avons développé un cadre sanitaire sur mesure, inspiré des concepts et définitions de l'Organisation mondiale de la Santé. Nous avons appliqué ce cadre afin de récolter des données sur les organismes publics et les lois relatives à la santé qui ont d'ores et déjà été édictées ou sont en cours d'élaboration, et avons comparé l'ampleur des transformations avant et après la Constitution de 2010. Notre analyse a révélé de multiples structures (lois et organes publics de mise en œuvre) réparties dans l'ensemble du système de santé, avec plusieurs nouvelles structures de gestion conformes à la décentralisation mais une fragmentation au niveau de la sous-fonction de régulation. En décomposant les fonctions normatives du système de santé, le cadre a permis d'établir une cartographie globale des différentes caractéristiques de ce système (fonctions, lois et organes de mise en œuvre). Nous sommes convaincus que notre cadre représente un outil utile pour les pays qui souhaitent développer et instaurer des bases juridiques propices à la création d'une couverture maladie universelle. La réforme constitutionnelle possède un pouvoir de mobilisation capable de faire progresser le changement institutionnel dans le domaine de la santé. Et ce, en renforçant deux aspects qui favorisent sa réalisation: l'acceptation de la part des intervenants, et l'autorité nécessaire pour agir.

## Resumen

La Constitución de Kenia de 2010 generó una serie de reformas en todos los sectores para ajustarse a los nuevos estándares constitucionales, incluida la transmisión y una amplia carta de derechos. La constitución representa una plataforma para promover los derechos sobre la salud y reestructurar los marcos jurídicos, institucionales y normativos con el fin de revertir las deficiencias crónicas y mejorar los resultados de la salud. Estas reformas de la salud, establecidas por mandato constitucional, son complejas. Asimismo, todas las áreas del sistema de salud se están transformando de manera simultánea, ya que se han promulgado varias leyes nuevas y se han establecido organismos de salud pública. Sin embargo, la falta de herramientas y métodos adecuados limitó la implementación de estos cambios tan complejos. Se elaboró un marco adaptado del sistema sanitario, que se guía por los conceptos y las definiciones de la Organización Mundial de la Salud, para tener una idea del alcance de las reformas sanitarias en los primeros 10 años de la constitución. En este contexto, se aplicó el marco para documentar las leyes sanitarias y los organismos públicos ya promulgados y en curso, en el que se comparó el grado de transformación antes y después de la Constitución de 2010. El análisis realizado reveló que se habían formado múltiples estructuras (leyes y organismos públicos de ejecución) en todo el sistema sanitario, que tenían muchas estructuras de gestión nuevas alineadas con la transmisión, pero que estaban fragmentadas dentro de la subfunción de reglamentación. Al desestructurar las funciones normativas del sistema sanitario, el marco permitió realizar un mapeo completo de los diversos atributos del sistema sanitario (funciones, leyes y organismos de ejecución). Se considera que el marco que se propone aquí es un instrumento útil para los países que quieren elaborar e implementar un fundamento jurídico propicio para la cobertura sanitaria universal. La reforma constitucional es una fuerza de movilización que permite obtener importantes avances en el cambio institucional del sector sanitario, lo que fomenta dos aspectos de la viabilidad del cambio: la aceptación de las partes interesadas y la autoridad para proceder.

## ملخص

أطلق الدستور الكيني لعام 2010 سلسلة من الإصلاحات عبر كل القطاعات للتوافق مع المعايير الدستورية الجديدة، بما في ذلك انتقال السلطة ووثيقة شاملة للحقوق. يعمل الدستور كمنصة للنهوض بالحقوق الصحية وإعادة هيكلة أطر العمل السياسية والقانونية والمؤسسية والتنظيمية بهدف رأب الفجوات المزمنة وتحسين النتائج الصحية. إن هذه الإصلاحات الصحية التي يفرضها الدستور تتسم بالتعقيد. تتغير جميع أطراف النظام الصحي بشكل متزامن، إلى جانب العديد من القوانين الجديدة التي تم سنها، وهيئات الصحة العامة التي تم إنشاؤها. وقد تعرض تنفيذ مثل هذا التغيير المعقد للتعويق بسبب الأدوات والأساليب غير الملائمة. للحصول على صورة لمدى الإصلاحات الصحية خلال السنوات العشر الأولى من تطبيق الدستور، قمنا بوضع إطار عمل معدّل للنظام الصحي، في ضوء مفاهيم وتعريفات منظمة الصحة العالمية. قمنا بتطبيق إطار عمل لتوثيق القوانين الصحية والهيئات العامة التي تم تنفيذها بالفعل، والتي هي قيد التنفيذ، وقمنا بمقارنة مدى التحول قبل وبعد دستور 2010. كشف تحليلنا عن العديد من الهياكل (القوانين والهيئات العامة المنفذة) التي تشكلت عبر النظام الصحي، مع العديد من هياكل الإشراف الجديدة المتوافقة مع انتقال السلطة، ولكن مع التجزئة داخل الوظيفة الفرعية التنظيمية. من خلال تفكيك وظائف النظام الصحي المعيارية، قام إطار العمل بتمكين رسم خرائط شاملة لمختلف سمات النظام الصحي (الوظائف والقوانين والهيئات المنفذة). نحن مؤمن بأن إطار عملنا هو أداة مفيدة للبلدان التي تريد وضع وتنفيذ أساس قانوني ملائم للتغطية الصحية الشاملة. الإصلاح الدستوري هو قوة دفع لتحقيق قفزات واسعة في التغيير المؤسسي الصحي، مما يعزز جانبين من جدوى التغيير: قبول أصحاب المصلحة وسلطة المضي قدما.

## 摘要

2010 年《肯尼亚宪法》推动了各部门的一系列改革，以遵循新宪法标准，包括权力下放和全面的人权法案。《宪法》为促进卫生权和调整政策、法律、体制和监管框架提供了一个平台，从而扭转长期差距，改善卫生成效。这些宪法规定的卫生改革很复杂。卫生体系的所有部门同时转型，颁布了若干新准则，并建立了公共卫生机构。此类复杂变革的实施受到了举措和方法不充足的阻碍。为了解该宪法前 10 年卫生改革的程度，我们根据世界卫生组织理念和界定，制定了一个合适的卫生体系框架。我们利用此框架记录已设立并正在实施的卫生法和公共机构，并对比了 2010 年《肯尼亚宪法》实施前后的改革程度。我们的研究结果表明，整个卫生体系形成多重结构（准则和实施中的公共机构），其中许多新的管理结构与权力下放一致，但在下级监管方面存在分歧。通过解析卫生体系标准职责，该框架提供了各种卫生体系属性的详细信息（职责，准则和实施机构）。我们认为，我们的框架有助于为某些国家实现全民健康覆盖打下坚实的法律基础并赋予实施。宪法改革可推动卫生体制取得突破性变革，增强了变革可行性的两个方面：利益相关者的接受程度和实施授权。

## Резюме

Конституция Кении от 2010 года инициировала комплекс реформ во всех секторах, чтобы привести их в соответствие с новыми конституционными стандартами, включая передачу полномочий и всеобъемлющий билль о правах. Конституция действует в качестве платформы для продвижения прав в области здравоохранения и реструктуризации политической, правовой, институциональной и нормативной базы для устранения хронических пробелов и улучшения результатов в отношении здоровья. Эти обусловленные конституцией реформы в сфере здравоохранения являются сложносоставными. Трансформация всех частей системы здравоохранения происходит одновременно, поэтому было принято несколько новых законов и были созданы органы общественного здравоохранения. Реализации таких сложных изменений препятствовали ненадлежащие инструменты и подходы. Для получения представления о масштабах реформ в сфере здравоохранения за первые 10 лет действия конституции авторы разработали адаптированную рамочную структуру для системы здравоохранения, руководствуясь концепциями и определениями Всемирной организации здравоохранения. Эту структуру применили, чтобы документально фиксировать, какие законы уже приняты и какие органы уже функционируют, а какие только находятся в процессе создания, и сравнили степень преобразований до и после принятия Конституции 2010 года. Как показал анализ, в системе здравоохранения существует множество структур (законов и исполнительных государственных органов), при этом большое количество новых надзорных структур связано с делегированием полномочий, но внутри регулятивной подфункции существует значительная раздробленность. Путем деконструкции нормативных функций системы здравоохранения рамочная структура позволила выполнить всеобъемлющее картирование различных атрибутов системы здравоохранения (функций, законов и исполнительных органов). Авторы считают, что такая рамочная структура является полезным инструментом для стран, которые хотят разработать и внедрить благоприятную правовую основу для всеобщего охвата услугами здравоохранения. Конституционная реформа — это движущая сила для значительного продвижения в институциональных изменениях в сфере здравоохранения, которая усиливает два аспекта осуществимости изменений: принятие заинтересованными сторонами и полномочия на выполнение.

## Introduction

The constitution of a country is its supreme law, which underpins all other laws as well as citizens’ pursuit of peace, justice and human development.^1^ Explicit constitutional provisions on the right to health exist in 28 of 47 Member States of the World Health Organization (WHO) African Region.^1^ Yet there is limited knowledge about country experiences with constitutionally mandated health reforms, particularly in low- and middle-income countries.

Kenya’s 2010 Constitution^2^ replaced the constitution adopted when the country gained independence in 1963, creating new normative, structural, institutional, policy and administrative standards. The 2010 Constitution provides important opportunities for fundamental reform, through key reform agents such as independent commissions and a restructured judiciary and parliament, among other core institutions, agencies and organs in government.^3^ A key constitutional standard requires the state to take policy, legislative and other measures to fulfil its obligations in respect of health. Consequently, in 2010 the Government of Kenya embarked on a reform of health policies, legislation and institutions. The health reforms are complex, with several multistakeholder processes running concurrently, developing various laws and detailing the formation or restructuring of various bodies. The reforms resonate with the United Nations high-level declaration on universal health coverage (UHC), which includes a commitment to strengthen legislative and regulatory frameworks for UHC.^4^ In this respect, measuring change in Kenya’s health reforms would contribute knowledge to advance UHC. 

On the 10th anniversary of the constitution, we describe our efforts to review the status of these health reforms. The Health Systems Governance Collaborative,^5^ in efforts to simplify governance to improve its understanding and applicability, has outlined a three-level approach for assessing the different elements and levels of governance: structural, process and outcome. Our paper focuses on structural measures, specifically the national laws and governance entities – the public implementing organizations and formal groupings across the entire health system. The aim of this article is to demonstrate an approach to measurement of health-system structure, and to apply that approach to analyse gaps and generate evidence for action to strengthen the structural capabilities in the Kenya health system. 

In the following sections we first outline our theoretical framework on structural reforms in health systems. We then describe the background to Kenya’s health-system reforms and the adapted health-system framework that we developed to analyse the multi-institutional reforms. Finally, we present our analysis and lessons learnt.

## Theoretical framework

There is considerable evidence associating the constitutional right to health with better health outcomes.^6^^,^^7^ A significant association has been found between a right to health in a national constitution and reductions in infant and under-five mortality rates.^6^ Other researchers found that institutional environments shaped by a right to health encourage more and better delivery of health services and can partly account for a positive impact on health outcomes.^7^ In this section we highlight some key linkages across health rights, health law, health institutions and health outcomes.

The rule of law is increasingly recognized as a determinant of health, and pivotal to health and development. WHO has observed that most public health challenges have a legal component and that the concept of public health law “includes the legal powers that are necessary for the State to discharge its obligation to realize the right to health for all members of the population.”^8^ Further, it has been argued that the rule of law is a largely unacknowledged prerequisite for a well-functioning health system.^9^ The law can translate vision into action on sustainable development, strengthen the governance of national and global health institutions and implement fair, evidence-based health interventions.^10^ The law can be an effective tool to harmonize the mandates of public agencies, clarify functions and promote multiagency cooperation; to designate the responsible agency to resolve a particular issue; and to create new entities to coordinate activities across multiple agencies.^10^ WHO notes that countries that have achieved UHC have built it on legal foundations, underscoring that developing and implementing a legal environment conducive to UHC is a critical investment.^11^ WHO highlights three critical elements to assess country contexts on whether UHC law reform is feasible: (i) whether there is acceptance of (or opposition to) the proposed reform; (ii) whether there is authority to proceed (especially authority from political decision-makers); and (iii) whether the country has the ability to complete the work (the capacity to make, implement and administer laws).^12^ Using the context of Kenya, we aim to demonstrate the extent of feasibility of UHC law reform, and to contribute lessons on the systematic assessment of legal and regulatory frameworks for UHC.

Effective health reforms should include reforming and restructuring the institutions through which health policies are implemented.^13^ One author has described institutions as the rules of the game – the formal and informal rules and norms that structure citizens’ rights, entitlements, opportunities and voices.^14^ A distinction can be drawn between organizations and institutions. Organizations (public or private) are created to perform defined functions. Organizations are primarily the agent for institutional change with the emphasis on the interaction between the rules of the game (institutions) and the players of the game (organizations).^14^ Formal institutions, the focus of this article, include the written constitution, laws, policies, rights and regulations enforced by official authorities (public organizations or agencies).^15^ An analysis of institutional change includes considering whether a particular function is necessary or not (for example, the need for an agency or new patterns of service delivery by organizations). Organizational change, however, focuses on internal capacities (for example, automation of business processes or upgrading equipment).^16^ Institutional change analysis must be driven by a focus on desired outcomes: in the case of health, multiple outcomes relating to UHC. Appropriate approaches and tools are needed to analyse and diagnose gaps and to predict further institutional change to strengthen the health system for UHC.^16^ We describe an approach to analyse concurrent change to multiple health laws and public organizations.

**Fig. 1:**
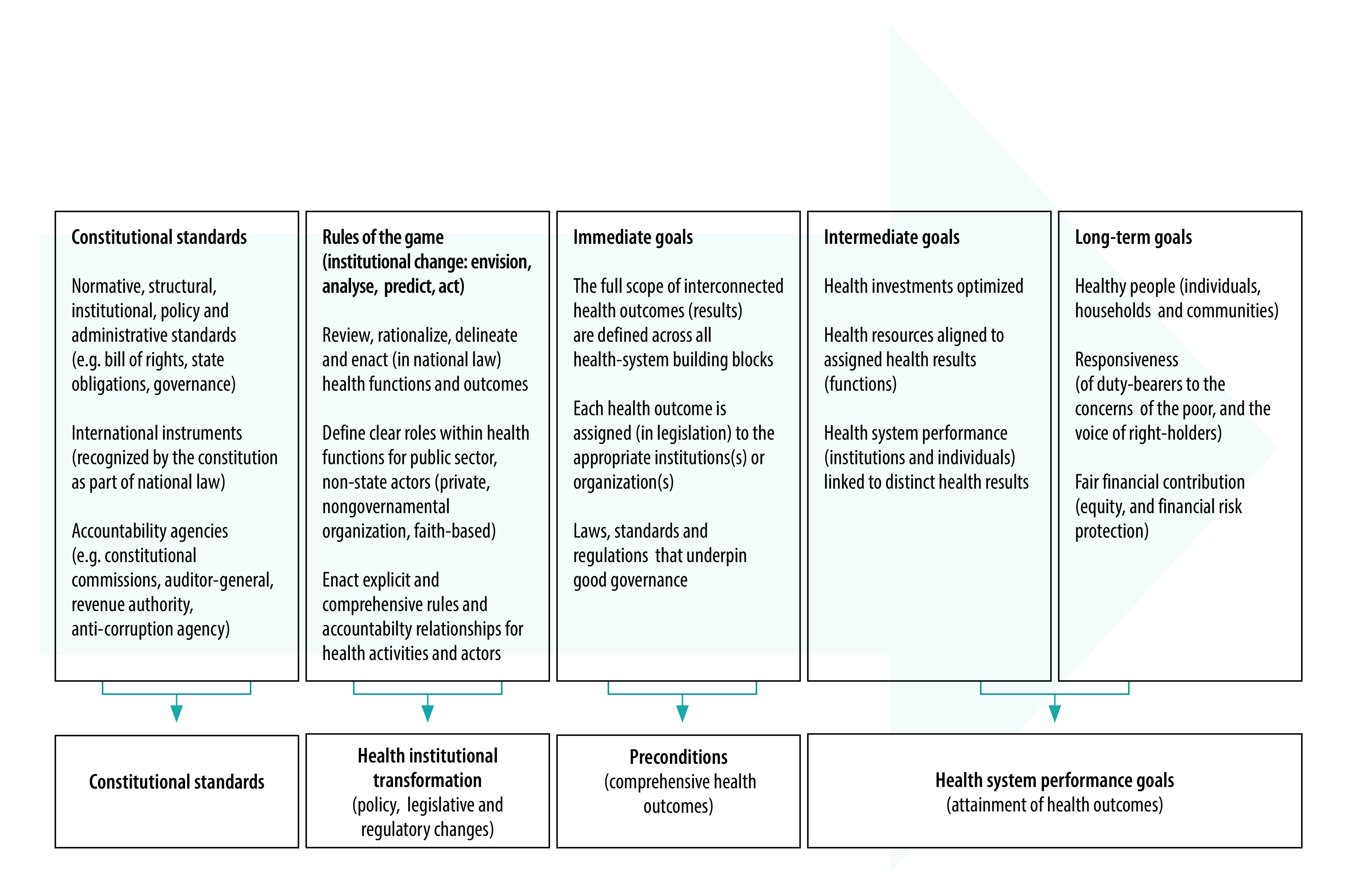
Theory of change on translating constitutional standards to health goals

We also consider social science theories related to advocacy and policy change efforts.^17^ Among these, the large-leaps theory posits that “when conditions are right, change can happen in sudden, large bursts that represent a significant departure from the past, as opposed to small incremental changes over time that usually do not reflect a radical change from the status quo.”^17^ In Kenya, the 2010 Constitution created a major shift in feasibility for health law reforms, which triggered large changes in policies, laws, institutional and regulatory frameworks. In Fig. 1, we illustrate a theoretical connection between constitutional standards and long-term health-system goals, via analysing institutional change, optimizing the interconnected health outcomes, and rationalizing their assignment to health actors (public and private). 

## Background to reforms

The key aspects of Kenya’s 2010 Constitution in relation to health were twofold: devolution of power to 47 county governments; and explicit provisions on the right to health. The extent of devolution of administrative functions varies across sectors. The health functions are extensively devolved: the national government is assigned health policy, national referral services and capacity-building for counties; county governments are assigned person-based and public health services within their jurisdictions.^18^^–^^20^ The constitution prescribed mechanisms and timelines for implementation of the various constitutional changes, including a time-limited independent body to oversee the transition to devolved government. This process entailed the development of enabling legislation and institutions for devolution, including intergovernmental relations, applicable to all sectors. The constitution triggered a large number of public-sector reforms and energized political commitment to reforms, including initiatives to streamline the governance of public agencies in all sectors, and to prioritize government investments and reforms in UHC, agriculture and nutrition, housing and manufacturing.^21^^,^^22^

**Fig. 2:**
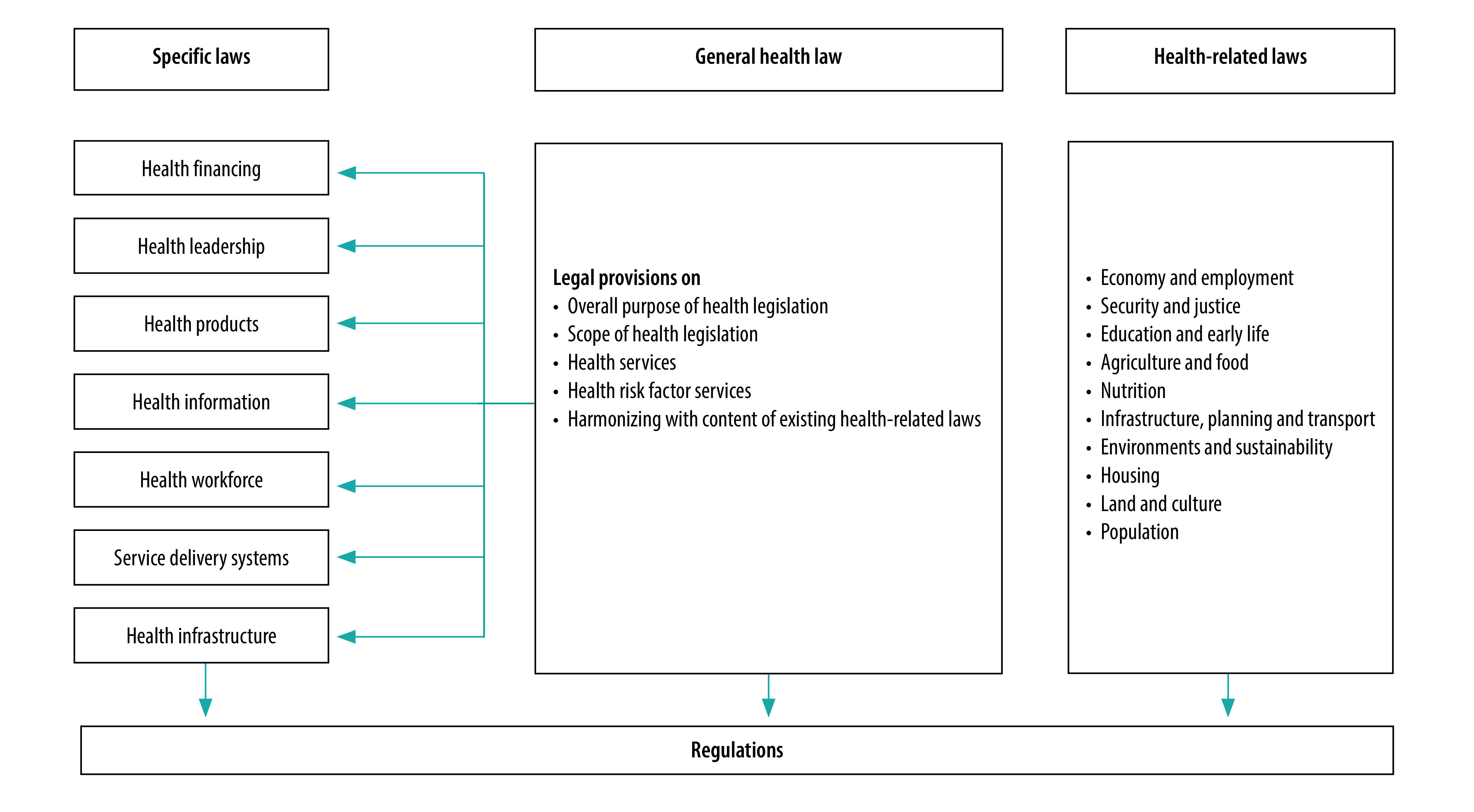
Comprehensive health legal framework for Kenya

Source: Kenya Health Policy (2012–2030).^23^

Notes: The Specific laws column is the same as the World Health Organization *Key components of a well functioning health system*^24^ with one additional component (Health infrastructure). After the onset of devolution, the health policy was subsequently updated to the Kenya Health Policy 2014–2030, which includes Health research – an eighth building block. For purposes of our analysis, our framework corresponds to the eight building blocks, and also separates Service delivery systems into two parts: person-based and population-based services (Table 1; Table 2).

To guide the transformation in the health sector, the Kenya Health Policy (2012–2030) was formulated^23^ with policy priorities structured around WHO’s six key components of a well-functioning health system: (i) leadership and governance; (ii) service delivery; (iii) health system financing; (iv) health workforce; (v) medical products, vaccines and technologies; and (vi) health information systems.^24^^–^^26^ This six-component structure was adapted for Kenya by highlighting additional policy issues and areas for investment. The policy proposed to overhaul the health legal framework by installing a new general health law and specific laws to restructure each component. This comprehensive legal framework incorporated health infrastructure as a seventh component (Fig. 2). After the county governments were elected in 2013, the health policy was validated and updated to the Kenya Health Policy (2014–2030),^27^ and health research was added as an eighth component. At various stages, the health ministry established ad hoc technical working groups and formal advisory panels. These groups act as the primary platforms for elaborating the needed change within the various reform initiatives and for facilitating broad stakeholder engagement and external technical support.^28^^,^^29^

## Conceptual and analytical framework

A major challenge in analysing the multi-institutional change in Kenya was the lack of a uniform and coherent approach. The use of simplistic tools to analyse complex health systems often contributes to interventions that upset the equilibrium of the system, which can lead to policy resistance from stakeholders.^30^ To align the health sector with the 2010 constitutional standards, Kenya’s health policy prescribed specific laws to transform multiple parts of the system, but lacked detail on the overall structural design, offering no rationale on the configuration of health functions or the implementing organizations envisioned to optimize health outcomes across the devolved system. Therefore, to analyse what has changed since 2010, we deconstructed the health legal (and institutional) framework, component-by-component and function-by-function, guided by WHO concepts and definitions.

WHO describes a health system as a set of interconnected parts that have to function together to be effective, consisting of all the organizations, institutions, resources and people whose primary purpose is to improve health.^31^^,^^32^ The WHO framework for health-systems performance assessment identifies four basic health-system functions through which health investments flow: (i) stewardship; (ii) resource generation; (iii) service provision; and (iv) financing. In this respect, a health system would be considered well performing when all the relevant organizations, institutions, resources and people are functioning together and contributing optimally to attaining three intrinsic goals or outcomes: health; responsiveness; and fair financial contribution.^33^ Consequently, health institutional reforms would be expected to optimize institutional capabilities to achieve the intrinsic health outcomes by transforming health functions component-by-component. 

We developed an approach – the adapted health-system framework – which enables a structured, all-inclusive framing of health functions, and promotes uniform and coherent analysis to identify structural gaps across the health system. We superimposed the core eight components of the Kenya Health Policy 2014–2030 and the four basic health-system functions described above. In this way, we created a grid with each cell representing a distinct health function. Our framework allows structure and function to converge, giving a perspective of the health system’s foundational elements and acting as a tool to visualize change. We used the framework to systematically document the national health laws and public bodies (those already enacted and those in progress) to assess the extent of change, diagnose gaps and identify corrective adjustments. Hence, this article is not concerned with monitoring constitutional implementation,^34^^,^^35^ or assessing whether specific health-system functions or accountability mechanisms are achieving desired outcomes (such as access to medicines^36^ or immunization coverage^37^).

Other authors have observed that stewardship is usually the most neglected function within health systems, yet it “anchors health to the wider society, comprising three broad tasks: providing vision and direction, collecting and using intelligence, and exerting influence through regulation and other means.”^38^ The sub-function of regulation has been discussed when describing the complex health-care regulatory system in the United States of America. Seven distinct areas of regulatory focus were identified,^39^ all addressing three competing health outcomes (access, quality and costs). These seven regulatory spheres are essentially a subset of our adapted framework since they relate to WHO’s concept of health stewardship, and they align with WHO’s six core health-system components. The spheres exclude health leadership (responsible for overall stewardship), and the other five components are subdivided and expanded to distinguish the perspectives relating to health regulation. Thus, health business relationships, public health and health research are distinct regulatory components. Our discussion will therefore highlight two stewardship sub-functions: overall system design and regulation.

## Assessment methods

We obtained empirical evidence for this assessment from two primary sources. First, all the authors were closely involved in the health reform processes in various capacities, either as government planning experts or as technical advisors, engaging through the technical working groups and advisory panels. Second, we analysed various documents including national policies, legislative instruments (laws, executive orders, legal notices and legislative bills). We identified all the instruments enacted for purposes relating to health, as published in the official Kenyan Government website.^40^ We then compiled a chronological list of these legislative instruments from 1921 to June 2020. For each instrument listed, we reviewed the legal text and identified two attributes: public body created and health function assigned. We then mapped all the bodies onto the adapted framework according to assigned function to see which governing entities and implementing organizations are in place and functional. We created two profiles: pre-constitution and post-constitution. Similarly, we mapped the initiatives that were in progress by June 2020 (technical working groups, advisory panels or parliamentary bills). To assess the extent of change in the regulatory sub-functions, we also extracted the data on the regulatory bodies formed to date (enacted and in-progress) and mapped these onto the seven regulatory spheres.^39^

## Legal and institutional changes

Before the 2010 Constitution, Kenya’s health system was managed centrally by two health ministries and governed through the Public Health Act of 1921 and other statutes governing specific functions. A total of 28 public bodies existed (in the statutes), although three of these were not currently operating, and we could not ascertain whether they had ever been constituted (Table 1). Shortcomings of the pre-constitution health structures were that institutional change was largely aligned to vertical public health programmes or to health professions. In particular, health professional bodies regulated most aspects of health in a cadre-centric model, creating a disproportionate focus on professional practice, with virtually no balancing laws or independent authorities to safeguard consumer interests (such as safety, pricing and confidentiality).

**Table 1:** Structure and function of public health bodies existing in Kenya before the 2010 Constitution, 1921–2010

Core components	Functions				Notes
	Stewardship: oversight	Financing: collecting, pooling and purchasing	Creating resources: investment and training	Delivering services: provision	
Leadership and governance	• Ministry of Medical Services (2008) • Ministry of Public Health and Sanitation (2008)	None	• Kenya Institute of Administration (1961)	None	The two health ministries were created as part of an expanded cabinet of the coalition government established after the signing of the Kenya National Dialogue and Reconciliation Accord (February 2008)
Health-system financing	None	• National Hospital Insurance Fund (1966)	None	None	NA
Health workforce	• 7 professional boards and councils, each established by statute: (i) Pharmacy and Poisons Board (1957); (ii) Medical Practitioners and Dentists Board (1978); (iii) Nursing Council of Kenya (1983); (iv) Radiation Protection Board (1984); (v) Clinical Officers Council (1989); (vi) Kenya Medical Laboratory Technicians and Technologists Board (2000); (vii) Council of the Institute of Nutritionists and Dieticians (2007)	None	• 7 university schools (various years): 4 medical schools 2 dentistry schools 1 pharmacy school • Kenya Medical Training College (1991)	None	The Pharmacy and Poisons Board is listed twice because it was established with a dual regulatory mandate from the outset (drugs and poisons, and pharmacy practice) Courses offered in the various medical schools are approved by the respective professional boards and councils: Kenyan Medical Practitioners and Dentists Council, and Pharmacy and Poisons Board
Service delivery (population-based)	• Central Board of Health (1921; not operational) • National Public Health Laboratory Service (1923) • Public Health (Standards) Board (1961; not operational) • Kenyan Board of Mental Health (1991; not operational) • National AIDS Control Council (1999) • HIV and AIDS Tribunal (2006) • Tobacco Control Board (2007)	None	None	None	The National Public Health Laboratory Service was created by the Ministry of Health, and is considered as a health ministry entity for administrative purposes
Service delivery (person-based)	None • The regulatory boards and councils oversaw their respective areas of practice within health facilities and undertook joint inspections	None	Various bodies • The referral hospitals are teaching facilities. Other public hospitals also act as learning centres for clinical training (pre-service and in-service)	Semi-autonomous referral hospitals: • Kenyatta National Hospital (1987) • Moi Teaching and Referral Hospital (1998) • Hospitals, health centres and dispensaries, managed centrally by health ministry	All public health-care facilities (except the two referral hospitals) were managed centrally by the two ministries of health. The Ministry of Public Health and Sanitation was responsible for rural health centres and dispensaries; and Ministry of Medical Services was responsible for hospitals. The health facilities were not established as distinct legal entities
Medical products and technology	• Pharmacy and Poisons Board (1957) • National Quality Control Laboratory (1992)	• Kenya Medical Supplies Agency (2000)	• Kenya National Blood Transfusion Service (2001)	None	Laboratory testing is one of the core functions of a national medicines regulatory authority. This overlap of roles between two bodies (Pharmacy and Poisons Board and National Quality Control Laboratory) contributes to conflicts in carrying out this regulatory function in Kenya The Kenya National Blood Transfusion Service was created by the Ministry of Health, and is considered as a public body for administrative purposes. The health law reforms captured a long-standing advocacy for the Kenya National Blood Transfusion Service to be established by statute
Health information systems	None	None	None	None	NA
Health infrastructure	None	None	None	None	NA
Health research	• National Council for Science and Technology (1977)	None	• Kenya Medical Research Institute (1979)	None	NA

NA: not applicable.

Notes: Cells of the adapted health-system framework show public health-sector bodies (and year of enactment) created before the 2010 Constitution of Kenya. Core components are based on World Health Organization’s (WHO) *Key components of a well functioning health system*, 2010.^24^ Functions are based on WHO’s framework for health systems performance assessment, 1999.^33^ In some cases we could not ascertain the reasons why a body was non-operational.

In the period since the 2010 Constitution was adopted there has been a large increase in the number of health bodies. This transformation has included enactment of eight laws and creation of 65 new bodies (16 national, two intergovernmental and 47 county health departments). Seven additional reforms were in progress by June 2020 (Table 2). 

**Table 2:** Structure and function of public health bodies created in Kenya after the 2010 Constitution, 2010–2020

Core components	Functions				Notes
	Stewardship: oversight	Financing: collecting, pooling and purchasing	Creating resources: investment and training	Delivering services: provision	
Leadership and governance	• Ministry of Health (2018) • Kenya Health Sector Intergovernmental Consultative Forum (2017) • 47 county health departments (2013) • Council of Governors Health Committee (2012)	None	• Kenya School of Government (2012), created by amalgamating the Kenya Institute of Administration and three other government training institutions	None	Three successive Executive Orders on the structure of the National Government (2013, 2016 and 2018) established a single health ministry and its portfolio responsibilities have not changed fundamentally
Health-system financing	• Independent body for health benefit package design, proposed by the Health Financing Reform Experts Panel, 2019 (in progress)	• Social Insurance Scheme to be created by converting the National Hospital Insurance Fund, proposed by the Health Financing Reform Experts Panel, 2019 (in progress)	None	None	The recommendations of the Health Financing Reform Experts Panel include the creation of a social insurance scheme, and two independent bodies: (i) health financing and (ii) health-care services accreditation
Health workforce	• Kenya Health Professions Oversight Authority (2017) • Kenya Health Workforce Council (2017) • Radiographers Board of Kenya (in progress) • 5 professional boards or councils, each established by statute: (i) Public Health Officers and Technicians Council (2013); (ii) Physiotherapy Council of Kenya (2014); (iii) Counsellors, Psychologists and Psychotherapists Board (2014); (iv) Health Records and Information Managers Board (2016); (v) Occupational Therapy Council of Kenya (2017)	None	• 4 university schools (various years): 4 medical schools 2 dentistry schools 1 pharmacy school • Kenya Medical Training College (1991)	None	The courses offered in the medical schools are approved by the respective professional bodies: Kenyan Medical Practitioners and Dentists Council, and Pharmacy and Poisons Board
Service delivery (population-based)	• National Cancer Institute (2012) • National Committee on Infant and Young Child Feeding (2012) • Health ministry technical working group on National Public Health Institute (in progress)	None	None	None	Population-based services are the focus of many donor-funded vertical programmes in Kenya’s health sector. Institutional change relating to public health tends to follow a similar pattern
Service delivery (person-based)	• Kenyan Medical Practitioners and Dentists Council (1978, revised 2019) • Health ministry technical working group on quality of care, addressing Health Act, 2017, Sect. 15(n), to provide for accreditation of health services, towards establishing an independent body for health services regulation, proposed by the Health Financing Reform Experts Panel, 2019 (in progress) • Health Benefit Package Advisory Panel (in progress) • Assisted Reproductive Technology Authority (in progress)	None	Various • The referral hospitals are also teaching facilities Other public hospitals also act as learning centres for clinical training, pre-service and in-service	• Kenyatta National Hospital (1987) • Moi Teaching and Referral Hospital (1998) • County health services	The role of the Kenyan Medical Practitioners and Dentists Council was expanded in 2019 to include regulation of health facilities. However, health services regulation (includes accreditation) is expected to transfer to a new independent body, in line with the Health Financing Reform Experts Panel recommendations (see above, under financing)
Medical products and technologies	• 2 parallel mechanisms, both addressing Part VII of the Health Act, 2017, single regulatory body for health products and technologies to be enacted: (i) health ministry technical working group on Kenya Food and Drugs Authority (in progress); (ii) Kenya Food and Drugs Authority Bill, 2019 (in progress)	• Kenya Medical Supplies Authority (2013) • Two parallel mechanisms in progress – both addressing Part XI of the Health Act, 2017 on Human Organs, Human Blood, Blood Products, Other Tissues and Gametes: (i) Draft Kenya National Blood Transfusion and Transplant Service Bill (2019, health ministry technical working group); (ii) Kenya National Blood Transfusion Service Bill (2020, parliament)	None	None	A proposed Kenya Food and Drug Authority is the anticipated single regulatory body for health products and technologies. Two parallel processes to create the proposed authority are in progress and need to be harmonized: one led by the health ministry, another led by parliament. Part XI of the Health Act, 2017 covers the full scope of human-derived medicinal products, but only provides for a blood service organization (Section 85). This discrepancy reflects in the scope of the two draft bills in progress, which need harmonizing
Health information systems	• Health ministry technical working group on e-health, addressing Health Act, 2017, Part XV – E-Health, Sect. 104(1), electronic health legislation to be enacted within 3 years (in progress)	None	None	None	A bill on electronic health has been drafted to implement the relevant provisions of the Health Act, 2017
Health infrastructure	• Independent body for health services regulation, proposed by the Health Financing Reform Experts Panel, 2019 (in progress)	None	None	None	Although the Kenya Health Policy distinguishes health infrastructure as a separate component, the regulation of health infrastructure is part of health services regulation
Health research	• National Commission for Science, Technology and Innovation (2013)	None	None	None	The National Commission for Science, Technology and Innovation is the successor to the National Council for Science and Technology (1977)

NA: not applicable.

Notes: Cells of the adapted health-system framework show public health-sector bodies (and year of enactment) created after the 2010 Constitution of Kenya up to June 2020. Core components are based on the World Health Organization’s (WHO) *Key components of a well functioning health system*, 2010.^24^ Functions are based on WHO’s framework for health systems performance assessment, 1999.^33^

Of the new laws, the Health Act, 2017 was the first major post-independence health legislation, delineating multiple health functions at the national, intergovernmental and county levels, establishing new bodies and mandating others to be enacted. The Act signalled a fundamental shift towards cadre-neutral health stewardship bodies (professions, products and institutions) and a greater focus on consumer aspects within health functions. These multiple reform initiatives demonstrate significant feasibility for health reforms. By prioritizing UHC reforms, political decision-makers have signalled authority to proceed, and broad acceptance by stakeholders. The multiple stakeholder engagement mechanisms led by the health ministry (technical working groups and advisory panels) enable the articulation of specific reforms within functions, facilitate consensus-building and isolate contentious issues to be resolved. Parliament is actively (but independently) engaged, including sponsoring bills in some priority areas (blood services, food and drug regulation), which creates pressure on health stakeholders to fast-track any related reform initiatives. These multiple forces are driving the large-leaps change to a new state of governance arrangements for health, aligned to devolution, and to broader government policies (such as governance of state agencies). 

The function of health stewardship has shown the greatest transformation, with the creation of a steward of stewards (the national health ministry) and delineated stewardship sub-functions across the devolved system. Of the 65 new bodies created, 59 have stewardship mandates (the other six are concerned with creating resources). Of the seven reforms in progress, six involve elaborating stewardship sub-functions (the other reform is concerned with a financing function). This considerable change would be expected to enhance system capabilities in providing vision and direction, collecting and using intelligence, and exerting influence, all contributing to the achievement of desired health outcomes. 

The seven distinct regulatory components are at varying stages of transformation (Table 3). Two new regulators have been formed (concerning health professionals and health research); two new regulators are mandated to be formed (for drugs and devices, and health-care institutions); three initiatives are in progress (concerning public health, financing arrangements and business relationships). However, two regulatory areas remain fragmented (public health and health-care professionals). For professions, five new cadre-centric bodies were created, resulting in a total number of 12 bodies (Table 2).

**Table 3:** Health regulatory bodies in Kenya, June 2020

Regulatory areas	Regulatory structures	
	Regulatory bodies (enacted or in progress)	Legal instruments^a^
Physicians and other health-care professionals	• Kenya Health Professions Oversight Authority	Health Act (2017), Sect. 60 (1)
	• 12 professional boards and councils (self-regulation)^b^	12 cadre-centric statutes (1957–2017)
Hospitals and other health-care institutions	• Kenya Medical Practitioners and Dentists Council	Amendment to the Medical Practitioners and Dentists Act (2019)
	• Proposed: independent mechanism for accreditation and quality assurance of health services (in progress)	Health Act (2017), Sect. 15(n); also recommended by the Health Financing Reform Experts Panel (2019)
Health-care finance	• Proposed: independent mechanism for health benefit package development (in progress)	Health Act (2017), Sect. 15(n); also recommended by the Health Financing Reform Experts Panel (2019)
Drugs and health-care products	• Pharmacy and Poisons Board	Pharmacy and Poisons Act (1957), Sect. 3
	• National Quality Control Laboratory	Pharmacy and Poisons Act (1957), Sect. 35D; amendment through Act No. 12 of 1992
	• Single regulatory body to be enacted (in progress)	Health Act (2017), Sect. 62; two Kenya Food and Drug Authority bills developed (health ministry, parliament); need harmonizing
Public health	• Central Board of Health (not operational)	Public Health Act (1921), Cap. 242
	• Public Health (Standards) Board (not operational)	Food, Drug and Chemical Substances Act (1965), Cap. 254
	• Tobacco Control Board	Tobacco Control Act, No. 4 (2007)
	• National Committee on Infant and Young Child Feeding	Breast Milk Substitutes Regulation and Control Act (2012)
	• Proposed: National Public Health Institute (in progress)	Draft National Public Health Institute Bill (2018)
Health-care business relationships	• Proposed: independent mechanism for health benefit package development and costing (in progress)	None
Funding of research	• National Health Research Committee	Health Act (2017), Sect. 93(1)

^a^ See also Table 1; Table 2.

^b^ A key recommendation of Kenya’s Presidential Task Force on Parastatal Reforms is the de-linking (from government ownership) of all bodies that are funded through members’ fees (member organizations) in all sectors. In the health sector, all the 12 cadre-centric boards and councils fall into this category, but the recommended de-linking has not yet been done.

Notes: We based the regulatory areas on the seven spheres of regulatory authority described by Field, 2007.^39^ The listed structures might not cover all the needed regulatory activities. In some cases we could not ascertain the reasons why a body was non-operational.

Overall, our analysis revealed structural gaps or inconsistencies across many health functions. We noted that, when the new laws and bodies were created, all the pre-constitution laws and bodies (including non-operational bodies) remained unchanged. Except for two merged health ministries, and minor amendments to other laws, these pre-existing structures were not eliminated or consolidated. The inherent fragmentation has therefore become entrenched in the system, with the attendant inefficiencies (gaps, duplication, overlaps and conflicts of mandates). A corrective action is therefore needed to rationalize and consolidate health functions, especially the regulation of public health and health-care professionals.

## Conclusion and lessons learnt

Our approach has enabled us to measure institutional change, diagnose gaps and generate evidence for predicting further change across the entire health system of Kenya. Overall, the multiple gaps identified across the health-system components demonstrate the multiple opportunities to streamline health functions across the system. To identify strategic options for further institutional change, a systematic review of the evidence is needed, function-by-function, focused on defined outcomes. However, because a national health system is one system with multiple interconnected parts, any predictions about change in one function require a holistic vision of the overall design of the health system, describing each distinct element, and how the various parts should operate together. By mapping backward from the overall health system goals, we need to define the desired outcomes relating to the distinct health functions, then identify actions that are needed to optimize these outcomes across the interconnected parts of the health system. 

We believe our adapted health-system framework is a useful tool for countries needing an all-inclusive framing of health-system structural elements to envision the overall design (future), analyse gaps (current) and predict the needed institutional change. In this respect, the grid is a versatile tool, to create context-specific frameworks, according to the health system attribute(s) mapped onto the cells (laws, bodies, gaps, outcomes). The various mappings can create multiple platforms for engagement, facilitating a holistic approach to health reforms.

The framework could be a useful tool for countries wishing to develop and implement a conducive legal environment for UHC. We have been able to quantify the extent of institutional change in Kenya’s health system and to diagnose gaps for corrective action to strengthen health functions, but we did not focus on the effects or impact of these changes. We encourage further studies to assess the adequacy of laws enacted and the capabilities or actual performance of the bodies created. We have learnt that a national constitutional reform is a mobilizing force for large-leaps institutional change in health, boosting two aspects of feasibility of conducting health reforms for UHC: acceptance by stakeholders; and authority to proceed from political decision-makers.^12^ The third aspect of feasibility – capability – requires capacity enhancement and interdisciplinary collaboration (health, legal and human rights), which promotes mutual learning and uniformity of actions. Priorities for capacity enhancement include technical framing of reform issues and formulating health law that is compliant with UHC. Implementing health institutional change requires a holistic, big-picture perspective, envisioning the overall health-system design as it should be, including the spatial arrangement of health functions and the corresponding outcomes. It is then possible to systematically analyse the structural elements to diagnose gaps and to predict change. 
